# Dr. Joseph H. Sutherland

**Published:** 1900-07

**Authors:** 


### OBITUARY.

Dr. Joseph H. Sutherland, of Niagara Falls, died at his home June 18, 1900, aged 59 years. For several weeks he had suffered from general debility. Dr. Sutherland was born in Canada on October 13, 1840. He attended the Toronto public schools. After receiving a common-school education he studied medicine and surgery. When the Civil War broke out he became a member of the hospital corps of the Confederate Army and served three years and six months. In 1865 he entered the University of Philadelphia and was graduated from that institution in 1867. He practised his profession and devoted some of his time to mining in California from 1867 to 1870. He next engaged in the oil business for six years in Oil Creek, Pa. He then removed to Butler County, Pa., and practised as a physician and surgeon. Afterward he spent some time in Mexico. In Washington, D.C., he made his home from 1888 to 1892. In the latter year he came to Niagara Falls, and lived there until his death. He was a member of the Board of Health for several years. He leaves a widow and two children.

